# Maternal carotid remodeling and increased carotid arterial stiffness in normal late-gestational pregnancy as assessed by radio-frequency ultrasound technique

**DOI:** 10.1186/1471-2393-13-122

**Published:** 2013-05-27

**Authors:** Li-Jun Yuan, Dan Xue, Yun-You Duan, Tie-Sheng Cao, Ning Zhou

**Affiliations:** 1 Department of Ultrasound Diagnostics, Tangdu Hospital, Fourth Military Medical University, Xi’an 710038, China

**Keywords:** Arterial stiffness, Intima-media thickness, Carotid artery, Normal pregnancy, Arterial remodeling

## Abstract

**Background:**

The adaption of elastic arteries to transient increase in hemodynamic load in normal pregnancy (NP) remains controversial. The purpose of this study was to investigate the NP carotid remodeling and regional arterial stiffness before and after parturition.

**Methods:**

Fifty-one NP women and 30 age-matched non-pregnant women were included. All women underwent right common carotid artery (RCCA) measurements with MylabTwice ultrasound instrument (Esaote, Italy). Carotid intima-medial thickness (IMT), pulse wave velocity (PWV, m/s), distensibility coefficient (DC, 1/KPa), α, β, augmentation index (AIx, %) and carotid arterial pressure were obtained by the newly developed ultrasound vascular wall tracking methods: automatic QAS (Quality Arterial Stiffness) and QIMT (Quality Intima-Medial Thickness) Follow up study was performed.

**Results:**

Compared to the non-pregnant controls, the arterial pressures were significantly increased and RCCA diameter was significantly enlarged in late gestational NP women. Twenty months after parturition, carotid diameter, DC, AIx, PWV and arterial wall tension were significantly decreased and had no significant difference with those in non-pregnant controls.

**Conclusions:**

Carotid arterial remodeling and stiffening could be seen in the normal pregnant women, which seems to be a physiological adaption and could be recovered post partum. QIMT and QAS together could provide a comprehensive assessment of the maternal carotid arterial changes during pregnancy.

## Background

It is crucial to understand the normal adaptations to pregnancy in order to understand the pathogenesis of the maternal disorders. The adaptions of heart to pregnancy have been well understood [1,2]. In contrast, the adaptions of arteries to this transient increase in hemodynamic load remain controversial. It has been shown that the systemic compliance increases during normal pregnancy, while, the study by Visontai Z et al. showed that the distensibility coefficient (DC) of the maternal carotid artery was decreased [3]. Mersich B et al. simultaneously measured aortic pulse wave velocity (PWV) and carotid artery elastic parameters, including DC, in a cohort of pregnant women. Interestingly, they found opposite changes occurred in carotid versus aortic stiffness during healthy human pregnancy: the aortic PWV decreased, signifying the increased compliance, while the carotid artery elastic parameters that related to carotid arterial compliance decreased [4]. To note, PWV has been accepted as the most simple, robust, and reproducible method to determine the arterial stiffness. Macedo ML et al. evaluated the carotid-radial and carotid-femoral PWV by applanation tonometry and found marginal difference between pregnant and non-pregnant women [5]. However, all above parameters were calculated from measurements of pulse transit time and the distance traveled by the pulse between two recording sites: carotid to radial or carotid to femoral, and thus could not be used to evaluate the local carotid arterial stiffness [6,7].

Recently, high resolution ultrasound acquisitions based on radio frequency signal gives the opportunity to assess precisely the local arterial wall properties [8], which could be performed by using the automatic QAS (Quality Arterial Stiffness) and QIMT (Quality Intima-Medial Thickness) packages provided by MylabTwice ultrasound instrument (Esaote, Italy). This newly developed ultrasound vascular wall tracking method has been validated and could give a rapid, delicate (resolution is 17 μm) and high reproducible estimate of IMT, and automatically measures the regional arterial stiffness. The purpose of this study was to investigate the regional carotid arterial stiffness during normal pregnancy by employing this new method, which might help for clarifying the controversies and further understanding of the maternal arterial adaptions to the transient increase in hemodynamic load.

## Methods

### General information

Fifty-one normal pregnant women with gestational age ≥28 weeks and 30 age-matched non-pregnant women were included. The menstrual cycle phase in these women was between 28 and 30 days. All pregnant women were nulliparas and had singleton pregnancies, and they were from the antenatal clinic of Tangdu Hospital, China between January 2010 and August 2012. The non-pregnant controls were from the outpatient department coming for physical examination during this period of time and volunteered for this study. They had no risk factors for arterial stiffness including hypertension, diabetes and hypercholesterolemia and they were lifetime non-smokers. All the subjects were asked to avoid caffeine or alcohol the night before the test. None of the included non-pregnant controls were taking any medicine during the study. All women took 10 minutes rest before the examination. The protocol was approved by the Human Subjects Ethics Committee of the Fourth Military Medical University. All women signed informed consent.

### Blood pressure measurement

Before ultrasound examination, brachial blood pressure (BP) measurements were taken using an oscillometric device (Collin) at 3-minute intervals for 20 minutes, and the average was taken as the casual BP level. BP measurements were performed by a single investigator (Dr. Xue) before noon in a quiet room with the subjects at a supine position and after 10 minutes rest. Mean arterial pressure (MAP) was calculated as diastolic BP + [(systolic BP-diastolic BP)/3].

### Carotid morphology and stiffness measurements by vascular ultrasound

These methods has been described before [9]. All women underwent the right common carotid artery (RCCA) measurements with MylabTwice ultrasound instrument (Esaote, Italy) equipped with Automatic QIMT (Quality Intima-Media Thickness) and QAS (Quality Arterial Stiffness) packages. Vascular probe LA523 with a frequency of 12 MHz was employed. All the measurements and calculations were done by two investigators (Drs. Xue &Yuan). Six consecutive measurements were performed and only when these consecutive 6 times measurements met the quality standard (Quality Control shown in green number on the screen during the scanning), was the average taken as the final result for this patient. These measurements and average calculations were automatically done and displayed on the left side of the sonogram.

*QIMT and carotid diameter measurements* were performed in the longitudinal way, strictly perpendicular to the ultrasound beam, with both walls clearly visualized. High-quality image was acquired along a minimum of 1.5 cm length of RCCA segment for reproducible measurements. The automatic QIMT calculation was activated and the thickness between the intima and the media on the standard B-mode image in real time was automatically measured using the radio-frequency reception signal. *QAS Measurements* automatically measured the modification of the arterial diameter between the systolic and diastolic phases on the same RCCA segments as in the measurement of IMT. Theoretically, carotid diameter waveforms were assessed by means of ultrasound and converted to carotid pressure waveforms using an empirically derived exponential relationship between pressure and arterial cross-section. The derived carotid pressure waveform is calibrated to brachial end diastolic and mean arterial pressure by iteratively changing the wall rigidity coefficient. This allows the calculation of the arterial stiffness [7]. Carotid stiffness indices: pulse wave velocity (PWV, m/s), distensibility coefficient (DC, 1/KPa), compliance coefficient (CC, mm^2^/kPa), α, β and augmentation index (AIx, %) were obtained.

Specifically, Pulse wave velocity (PWV) was calculated from the following equation: $\mathrm{PWV}=\frac{1}{\sqrt{\rho \cdot \mathrm{DC}}}=\sqrt{\frac{D^{2}\cdot \mathrm{\Delta p}}{\rho \cdot \left(2\cdot D\cdot \mathrm{\Delta D}+\Delta D^{2}\right)}}$, where, D: Diastolic diameter; ΔD: Change of diameter in systole; DC: Distensibility coefficient; Δp: Local pulse pressure; ρ: Blood density. PWV is a functional parameter directly affected by arterial wall stiffness. Due to the extensive data linking PWV to the risk of incident cardiovascular events, PWV is regarded as the in vivo “gold standard” index of arterial stiffness.

Distensibility Coefficient (DC) is the fractional change in cross-sectional area relative to the change in arterial pressure. DC was calculated as: $\mathrm{DC}=\frac{\mathrm{\Delta A}}{A\cdot \mathrm{\Delta p}}=\frac{2\cdot D\cdot \mathrm{\Delta D}+\Delta D^{2}}{D^{2}\cdot \mathrm{\Delta p}}$, where A: Diastolic area; ΔA: Change of area in systole; D: Diastolic diameter; ΔD: Change of diameter in systole; Δp: Local pulse pressure; Compliance Coefficient (CC) is calculated as: CC = ΔDπDs/2ΔP, where D_S_:diameter in systole; Stiffness index β was expressed as ln (SP/DP) × D/ΔD, where SP and DP are carotid systolic and diastolic pressure respectively. P_T1_ might not be an independent hemodynamic variable, though it would be augmented due to increased PWV and the return of reflected waves to the heart from the periphery.

Augmentation index (AIx) is another parameter that could be obtained from pressure measurements at a single site. It is calculated as the difference between the second and the first systolic peaks observed on the arterial waveforms, and it is expressed as a percentage of the pulse pressure (AIx = [AP/(Loc Psys – Loc Pdia)] × 100, where, Loc Psys: Local pressure – systolic; Loc Pdia: Local pressure – diastolic. AP: augmented pressure), and is regarded as an indirect measure of arterial stiffness and wave reflection.

In addition to these morphological and stiffness parameters, the resistance index (RI) and the pulsatility index (PI) of the right carotid artery were also acquired by traditional vascular ultrasound. RI = (maximal flow velocity-minimal flow velocity)/maximal flow velocity and PI = (maximal flow velocity-minimal flow velocity)/mean flow velocity.

### Statistical analysis

All values are expressed as mean ± SD for continuous variables. As gestational age, body mass index, heart rate (HR) and blood pressure could have affected the measurements, the results for carotid arterial diameter and arterial stiffness parameters of PWV, DC, CC, α and β were adjusted for these covariates, using a general linear model. Group profiles were compared with paired or unpaired Student’s t-test. Correlations between arterial parameters and carotid pressures were analyzed with Pearson’s correlation coefficient (r). A p value < 0.05 was considered a statistically significant difference. The statistical software package SPSS 12.0 (SPSS Inc., Chicago, IL) was used for all data analyses. Reproducibility and variability have been performed before and good agreement and correlation were observed between measurements taken by the same observer and the two different observers [9].

## Results

### General information of the populations

Recordings were successfully obtained from all women. The demographic characteristics, brachial and carotid pressures of the women participating in the study were given in Table 1. The heart rate (HR), systolic and mean arterial blood pressures were significantly higher in late gestational pregnant women compared to the non-pregnant controls (all p < 0.0001).

**Table 1:** Demographic characteristics and carotid pressures of the study population

	**Non-pregnancy**	**Normal pregnancy**	***P Value***
	**(n = 30)**	**(n = 51)**	
MA, years	25.2 ± 2.6	26.2 ± 3.8	0.214
GA, weeks	-	34.0 ± 3.8	-
BMI, kg/m^2^	18.6 ± 1.3	25.2 ± 2.8	<0.0001
HR, bpm	72 ± 12	94 ± 15	<0.0001
Brachial SP, mmHg	97.9 ± 8.6	109.7 ± 12.7	0.0001
Brachial DP, mmHg	71.2 ± 8.2	75.0 ± 7.9	0.367
Brachial MAP, mmHg	65.9 ± 5.6	86.6 ± 8.5	<0.0001
Carotid SP, mmHg	97.9 ± 8.7	106.9 ± 10.1	<0.0001
Carotid DP, mmHg	71.1 ± 8.3	74.3 ± 9.7	0.138
Carotid MAP, mmHg	66.9 ± 5.9	85.1 ± 8.8	<0.0001

Notes: Values are given as mean ± SD. *MA* Maternal age, *GA* Gestational age, *BMI* Body mass index, *HR* Heart rate, *SP* Carotid systolic pressure, *DP* Carotid diastolic pressure, *MAP* Mean arterial pressure.

### The carotid arterial morphological and hemodynamic remodeling

The morphological changes of RCCA in these women were listed in Table 2. Compared to the non-pregnant controls, the diameter of RCCA was significantly enlarged (p < 0.0001) and RI and PI of RCCA were significantly decreased in normal pregnancy (p = 0.007, 0.006). However, these differences were not existed after adjusted for gestational week, BMI, HR and carotid pressures. No significant alteration of IMT was seen in normal pregnancy in comparison with non-pregnant controls (p = 0.204).

**Table 2:** Right common carotid arterial characteristics of the study population

	**Non-pregnancy**	**Normal pregnancy**	***P Value***	***P Value***^***1***^
	**(n = 30)**	**(n = 51)**		
IMT, μm	392 ± 85	369 ± 78	0.204	0.497
D (mm)	6.3 ± 0.6	7.1 ± 0.4	<0.0001	0.399
RI	0.78 ± 0.05	0.73 ± 0.08	0.007	0.521
PI	2.13 ± 0.36	1.84 ± 0.41	0.006	0.270
DC, 1/kPa	0.05 ± 0.02	0.04 ± 0.02	0.0003	0.200
CC, mm^2^/kPa	1.33 ± 0.74	1.05 ± 0.41	0.048	0.101
α	2.73 ± 1.05	3.50 ± 1.18	0.004	0.046
β	5.62 ± 2.11	7.57 ± 3.50	0.004	0.046
PWV, m/s	5.29 ± 1.08	6.38 ± 1.35	0.0003	0.067
AIx, %	2.32 ± 5.87	-4.29 ± 6.48	<0.0001	0.440

Notes: Values are given as mean ± SD. *IMT* Intima-media thickness, *RI* Resistance index, *PI* Pulsatility index, *DC* Distensibility coefficient, *CC* Compliance coefficient, *α and β* Arterial stiffness, *PWV* Pulse wave velocity, *AIx* Augmentation index. P Value^1^, P value adjusted for body mass index, gestational weeks, heart rate, systolic pressure, diastolic pressure and mean arterial pressure.

### The carotid arterial stiffness remodeling

The arterial stiffness changes of RCCA in these women were listed in Table 2. The carotid arterial distensibility coefficient (DC) was significantly decreased in normal pregnancy compared to non-pregnant control group (p = 0.0003). Accordingly, arterial stiffness parameters α and β were significantly increased in pregnant women (p = 0.004, 0.004). AIx was significantly lower in pregnant women compared to non-pregnant women (p < 0.0001). PWV was remarkably faster in pregnant women compared to non-pregnant controls (p = 0.0003). Carotid wall tension was significantly increased compared to non-pregnant controls (38.6 ± 5.4 mmHg/cm vs. 32.9 ± 4.8 mmHg/cm, p < 0.0001). Slight but significant difference was still existed in α and β between NP and non-pregnant controls after adjusting for gestational week, BMI, HR and carotid pressures (Table 2) (p = 0.046, 0.046).

### Correlation of arterial stiffness parameters with arterial pressure

**Figure 1:**
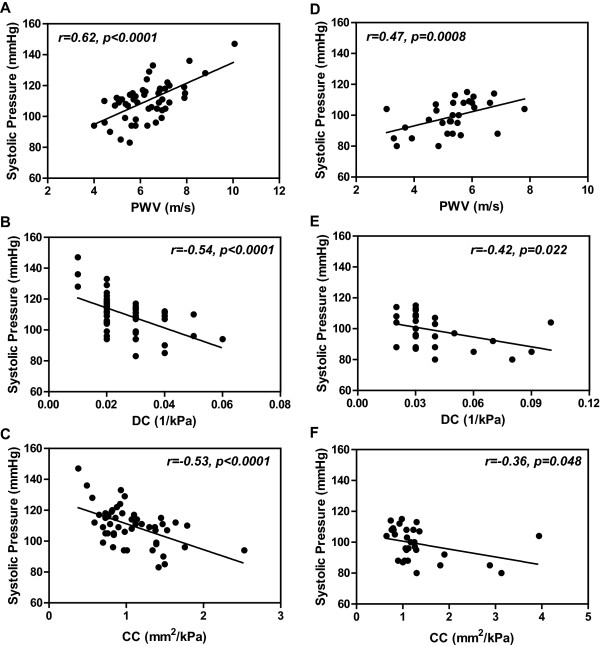
**Correlations of carotid arterial PWV, DC and CC with carotid systolic pressure in normal pregnant women (A-C) and non-pregnant controls (D-F).** PWV, pulse wave velocity; DC, distensibility coefficient; CC, compliance coefficient.

**Figure 2:**
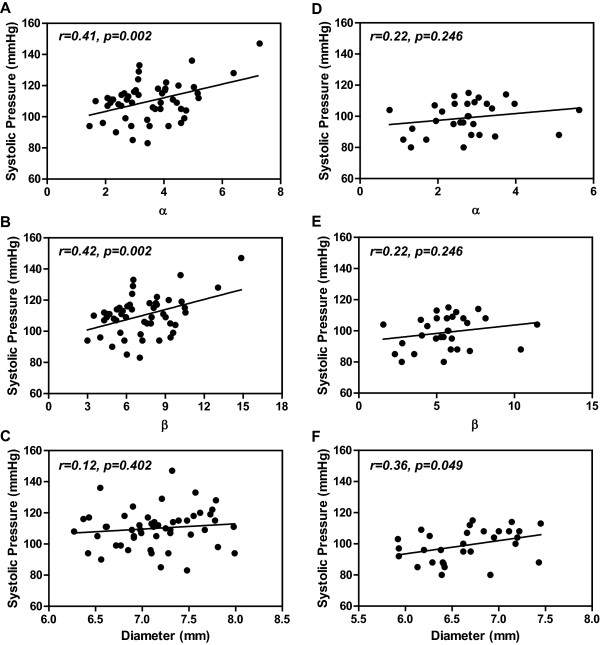
**Correlations of carotid arterial α, β and internal diameter with carotid systolic pressure in normal pregnant women (A-C) and non-pregnant controls (D-F).** α and β, arterial stiffness.

Arterial PWV, DC and CC correlated closely with carotid arterial systolic pressure both in the normal pregnant women and non-pregnant controls (Figure 1), while α and β correlated closely with the carotid arterial pressure only in the normal pregnant group (Figure 2).

### Follow up study

All pregnant women had natural baby delivery and normal outcome with no complications postpartum. Twenty months after parturition, carotid diameter, DC, PWV, α and β and wall tension was significantly decreased and had no significant difference with those of non-pregnant women (Table 3).

**Table 3:** Comparisons of carotid parameters before and 20 months postpartum

**Parameters**	**Normal pregnancy**	**Normal pregnancy**	**Non-pregnancy**
	**(During pregnancy) (n = 12)**	**Postpartum (n = 12)**	**(n = 15)**
Carotid IMT (μm)	326 ± 85	399 ± 63	407 ± 84
Carotid Diameter (mm)	7.38 ± 0.29	6.87 ± 0.25^*^	6.53 ± 0.39
Carotid SP (mmHg)	111 ± 14	100 ± 10^*^	101 ± 10
Carotid DP (mmHg)	75 ± 8	77 ± 8	73 ± 7
Carotid MAP (mmHg)	87 ± 9	85 ± 8	76 ± 11
DC (1/kPa)	0.02 ± 0.01	0.04 ± 0.01^*^	0.03 ± 0.01
PT1 (mmHg)	109 ± 3	99 ± 10	99 ± 12
PWV (m/s)	6.4 ± 0.9	4.9 ± 0.8^**†**^	5.4 ± 0.6
AIx (%)	-2.64 ± 2.63	0.32 ± 8.78	0.16 ± 2.99
Tension (mmHg/cm)	41.2 ± 5.0	34.3 ± 0.1^*^	33.2 ± 4.6

Notes: All abbreviations were the same as in Table 2. ^*^Compared to those before pregnancy, p < 0.01; ^†^Compared to those before pregnancy, p < 0.05.

## Discussion

The study has demonstrated that the regional carotid arterial stiffness as well as internal diameter significantly increases, and the resistance index and pulsatility index decreases in normal late gestational pregnant women compared to age-matched non-pregnant controls. Slight but significant difference was still existed in α and β between NP and non-pregnant controls after adjusting for gestational week, body mass index, heart rate and carotid pressures. All these changes could be recovered twenty months postpartum. Carotid PWV, DC, CC, α, β were all closely correlated with carotid systolic and mean arterial pressures in normal pregnant women.

Animal experimental studies have shown that increases in blood flow due to an arteriovenous shunt would increase vessel diameter [10]. Based on this finding, the increased carotid arterial diameter in pregnancy might be result of the increased carotid blood flow, attributed by the increased cardiac output. No significant difference was seen in intima-media thickness (IMT) between normal pregnant and non-pregnant women, which is in consist with the previous study [4]. However, acute changes in IMT in response to acute blood pressure and vascular tone modifications have been noticed [11].

Arterial stiffness is an independent marker for increased risk of cardiovascular complications. Studies in non-pregnant populations have demonstrated that arterial stiffness is increased in patients with diseases like hypertension, diabetes mellitus [12,13]. In addition, in one of our previous studies in pregnancy showed that preeclampsia is characterized by increased maternal arterial stiffness even after the adjustment for maternal body mass index and arterial pressure [9]. PWV is currently considered the most useful and robust index of arterial stiffness [14]. In the current study, it demonstrated that PWV significantly increased in the normal pregnant women, indicating increased arterial stiffness in these populations. Though both DC and CC decreased in normal pregnancy, DC seems a better parameter identifying changed arterial compliance. AIx is a a composite measure of systemic arterial stiffness and wave-reflection amplitude [15]. AIx was significantly lower in pregnant than non-pregnant women shown in the current study. In addition to the carotid arterial stiffness parameters, the arterial resistance and pulsatile indices were all decreased, which might contribute to the increase of maternal cerebral blood flow during normal pregnancy reported by Nevo O et al [16]. However, all these differences were not existed after adjusting for maternal BMI, gestational age, carotid pressures and HR, except α and β, where marginal difference was shown. This strongly indicate that these differences in carotid arterial morphology, hemodynamics and stiffness might be a reflection of the combined influences of BMI, HR, and carotid pressures on these parameters, not reflecting a true change in these women. The controversies in literatures regarding the arterial stiffness changes in normal pregnant women might be related to the fact that whether these indices have been adjusted to the maternal body mass index and arterial pressure. Twenty months postpartum, carotid arterial diameter and arterial stiffness parameters were all returned to normal. These changes further indicate that the carotid arterial remodeling is a normal and recoverable physiological adaption to pregnancy.

It has to note that all the sujected in this sutdy adopted a supine position for the examination, which may have a substantial impact on cardiovascular parameters in late gestation, as uterine compression of the inferior vena cava can reduce return of blood to the heart.

## Conclusion

Carotid arterial remodeling and stiffening could be seen in the normal pregnant women, which seems to be a physiological adaption and could be recovered postpartum. QIMT and QAS together could provide a comprehensive assessment of the maternal carotid arterial changes during pregnancy.

## Competing interest

The authors declared that they have no competing interests.

## Authors’ contributions

The contributions of individual authors to this paper were as follows. Dr. YLJ, XD, DYY participated in 1. The conception and design, acquisition, analysis and interpretation of data, development of the hypothesis and research plan, establishment of methodology; 2. Drafting of the manuscript and critical revision of the manuscript for intellectual content and 3. Final approval of the version to be published. CTS and ZN involved in 1. Acquisition of data, analysis and interpretation of data; 2. Assistance with revising the manuscript and 3. Final approval of the version to be published. All authors read and approved the final manuscript.

## Pre-publication history

The pre-publication history for this paper can be accessed here:

http://www.biomedcentral.com/1471-2393/13/122/prepub
